# An analysis of signal processing algorithm performance for cortical intrinsic optical signal imaging and strategies for algorithm selection

**DOI:** 10.1038/s41598-017-06864-y

**Published:** 2017-08-03

**Authors:** J. A. Turley, K. Zalewska, M. Nilsson, F. R. Walker, S. J. Johnson

**Affiliations:** 1 School of Electrical Engineering and Computer Science, University of Newcastle, Callaghan, NSW Australia; 2 School of Biomedical Sciences and Pharmacy and the Centre for Translational Neuroscience and Mental Health Research, University of Newcastle, Callaghan, NSW Australia; 3 Hunter Medical Research Institute, Newcastle, NSW Australia

## Abstract

Intrinsic Optical Signal (IOS) imaging has been used extensively to examine activity-related changes within the cerebral cortex. A significant technical challenge with IOS imaging is the presence of large noise, artefact components and periodic interference. Signal processing is therefore important in obtaining quality IOS imaging results. Several signal processing techniques have been deployed, however, the performance of these approaches for IOS imaging has never been directly compared. The current study aims to compare signal processing techniques that can be used when quantifying stimuli-response IOS imaging data. Data were gathered from the somatosensory cortex of mice following piezoelectric stimulation of the hindlimb. The effectiveness of each technique to remove noise and extract the IOS signal was compared for both spatial and temporal responses. Careful analysis of the advantages and disadvantages of each method were carried out to inform the choice of signal processing for IOS imaging. We conclude that spatial Gaussian filtering is the most effective choices for improving the spatial IOS response, whilst temporal low pass and bandpass filtering produce the best results for producing temporal responses when periodic stimuli are an option. Global signal regression and truncated difference also work well and do not require periodic stimuli.

## Introduction

Optical imaging techniques have proven to be a useful tool in understanding changes in brain function. Intrinsic Optical Signal (IOS) imaging uses light in the visible spectrum to investigate activity dependent changes within the cerebral cortex^1–4^. IOS imaging has been most commonly used to examine changes in levels of oxygenated haemoglobin (HbO_2_) and deoxygenated haemoglobin (HbR). First developed by Grinvald *et al*.^5^, IOS is gaining increasing popularity, particularly when imaging haemodynamic responses in very small animals, such as mice, due to modest hardware costs involved in comparison to other techniques such as micro-CT and MRI^6^. The simplicity of the technique also allows IOS imaging to be combined with other imaging or data acquisition modalities.

When the brain is stimulated by an external source, for example, tapping on the hind paw of a mouse, a haemodynamic response is triggered, in this case, within the somatosensory cortex^7^. This response produces a change in haemoglobin concentration, as well as blood flow and blood volume^8, 9^. This alters the absorption properties within the tissue, affecting the way light behaves as it passes through it^10^. Using this property, the brain can be illuminated with visible light and a camera placed above to image the surface. Any changes in the way the light scatters or is absorbed can be picked up by these images. The IOS response is predictable and repeatable, however, an uncertainty exists in how these haemoglobin changes relate to neural activity^11, 12^.

IOS imaging typically involves several seconds of baseline imaging, where the brain is imaged in its resting state with no stimuli. These frames are averaged together to create a single baseline image. Imaging continues as the stimulus is applied and the response is captured for a period of time afterwards, usually between 1.5 and 40 seconds. Depending on the wavelength of illumination chosen, different information can be obtained from these results. This is due to the different absorption spectra for both HbO_2_ and HbR. For light in the red region (around 630 nm), HbR has significantly higher absorption properties than that of HbO_2_, thus the results obtained from this wavelength correlate highly to changes in HbR. Selecting an isobestic point, such as 530 nm, weights the changes for both HbO_2_ and HbR equally, allowing visualisation of the total change in haemoglobin (HbT)^13^. This process can be taken a step further by imaging whilst illuminating with a larger number of wavelengths separately, and combining the data using least squares and the modified Beer-Lambert law^14^. This produces a space-time matrix containing the absolute changes in concentrations of HbR and HbO_2_. Depending on the focus of the research being carried out, this output is usually viewed as either a spatial response map^15^ or a temporal^16^ haemodynamic response.

Implementing a multi-spectral setup during IOS brain imaging involves cycling between the different wavelengths of illumination between each frame of image capture. This is typically done with a ring of timed light emitting diodes (LEDs)^17, 18^ or a filter wheel^19, 20^ with different wavelengths. The collected imaging data will therefore have a single wavelength for each frame.

Mice offer several advantages over rats during IOS imaging. Their skull is thinner which means invasive procedures such as a craniotomy need not be performed to gain visibility of the brain’s surface. Even though mice are smaller, decreasing the negative effects of scattering, they require more accurate and specialized equipment, which has a detrimental effect on the signal-to-noise ratio. These issues, combined with the shorter and noisier response of the mouse, means image and signal processing methods are critically important.

One of the main challenges facing IOS imaging is separating the signal of interest from multiple sources of disturbance. These unwanted contributors to the IOS imaging signals include periodic sources of interference, such as breathing and heartbeat, and noise from random illumination fluctuations, camera noise created during image collection^21^ and inter-trial variability^22^. Other sources of disturbances include artefacts from non-stimuli related brain activity and changes to the brain window during imaging. It is beneficial to quantify the disturbances of the system so more appropriate signal processing can be performed for the specific setup. It should be noted that some of these features will be unique to our imaging setup, with electrical noise varying with different camera and illumination setups, and biological disturbances changing with different anaesthesia and animal species. In most cases basic signal processing techniques are applied to IOS imaging data to improve the signal-to-noise ratio. To deal with the added disturbances and decreased data quality obtained when imaging mice, more advanced and specialized processing needs to be implemented^23^.

A wide range of techniques has been used in an attempt to tackle the specialised issues involved with intrinsic optical signal imaging. Although every research group will face slightly different concerns depending on their individual interest and setup, the general concepts involved with the signal processing are consistent across techniques. At a minimum, averaging over trials is typically carried out on a repeated number of stimulus trials^21, 24^. However, there are many other signal processing routines that are available and have been applied to IOS data, which may provide superior performance over averaging. These include temporal bandpass filtering^25, 26^, temporal low pass filtering^27, 28^, spatial Gaussian filtering^18, 29^, principal component analysis^30, 31^, global signal regression^25^, Bayesian priors^32^ and temporal encoded mapping^28^. Principal component analysis has several variants including indicator function^30, 33, 34^, truncated difference^30^ and extended spatial decorrelation^31^.

Several research groups have previously performed their own comparisons of post-processing techniques, however typically as a single, or small number of techniques compared to the standard averaging. Often qualitative comparisons are made, where quantitative would be more beneficial, and typically for the visual cortex. A full quantitative comparison between all the techniques on real data has not yet been carried out.

Previously, Kalatsky and Stryker^28^ highlighted the advantages of temporal encoded mapping over averaging by comparing the results of each to a reference map of the visual cortex of the cat. Stetter *et al*.^31^ made a qualitative comparison of ESD and PCA applied to data from the rat and ferret visual cortex. Everson *et al*.^33^ compared two variations of indicator function. An artificial visual response was layered over real macaque imaging data and the resulting indicator function’s result was compared to this response to produce a quantifiable performance for both methods. Gabbay *et al*.^30^ also used an artificial visual response layered over resting data of the macaque to concluded that truncated difference is more sensitive than indicator function, whilst both performed better than averaging alone. Yokoo *et al*.^35^ used multi-condition visual stimuli to compare pairwise truncated difference to several indicator function based techniques, namely pairwise indicator function, canonical variate analysis and generalized indicator function. A synthetic visual response was added to a cat’s real non-response imaging data and each functions similarity to the synthetic response was calculated, as well as the resulting signal to noise ratio. For this case, generalized indicator function had superior performance to canonical variate analysis. The techniques were also compared for real cat imaging data and it was found that for visual stimuli, generalized indicator function produced qualitatively similar results to the other techniques however required less subjective intervention.

The general consensus is that there are very many methods which can produce better results than averaging alone, at least for some types of data, although it is not yet clear what features of the data make it suitable for which method. Of the PCA based methods the results to date suggest that truncated differences or perhaps indicator function are the most effective^30, 34^, at least for visual cortex data, although results vary depending on the quality of the data.

In this paper we have compared the most common and relevant options currently being performed: averaging over trials, spatial Gaussian filtering, temporal low pass filtering, temporal band pass filtering, global signal regression, principal component analysis and truncated differences. The effectiveness of each technique’s ability to remove noise and extract the IOS signal is compared for both spatial and temporal responses on real IOS data, both quantitatively and qualitatively. The results are also compared over a varying number of stimuli trials, enabling more flexibility when selecting the appropriate signal processing technique. Since each imaging setup encounters different types of noise in varying magnitudes, careful analysis of the advantages and disadvantages of each method is presented such that the best choice can be made for a given IOS imaging setup.

## Methods

### Experimental Setup and Imaging Procedure

A group of 23 C57BL/6 adult male mice was used for imaging, obtained from the Animal Services Unit at the University of Newcastle. The experiment was approved by the University of Newcastle Animal Care and Ethics Committee and conducted in accordance with the New South Wales Animals Research Act (1985) and the Australian Code of Practice for the use of animals for scientific purposes.

Under anaesthesia and before imaging, a thin film of agarose (type IX ultra-low gelling temperature) was layered on the surface of the skull, a glass coverslip placed on top, creating a clear, imaging window^36^. A physical stimulus was applied to the hind paw using a piezo-electric actuator and a miniature piezo driver (PDu100B, PiezoDrive). The piezo was deflected against the paw with a frequency of 10 Hz for the duration of the 1.5 s stimuli period. The imaging hardware setup consisted of a CCD camera (GenieM640, Teledyna Dalsa) coupled with a custom macro-lens. The surface of the brain was illuminated with a custom-made LED ring consisting of four wavelengths: 470 nm, 530 nm, 588 nm and 627 nm. (Luxeon Star LEDs)^6^. The LEDs had switching times within the nano-second range which is therefore not an issue for the imaging frequency we are using.

One session of imaging consisted of continuous recording of 100 trials. For each animal, three imaging sessions were recorded and the session with the strongest signal kept for analysis. This chosen session was then split into two halves with equal number of trials. The processing techniques were performed separately on each half. (This was done to allow for signal-to-noise estimations.) To consider the performance of the techniques as the number of trials changes, the first N trials from each half were processed, where N = 1, 10, 25, 50. Each trial lasted 10 seconds and consisted of the first 1.5 s of baseline imaging, followed by 1.5 s of hind paw stimulation with imaging, followed by 7 s of post stimuli imaging. The camera was set to 60 frames per second, cycling through the four wavelengths of illumination between each frame (15 frames per second each wavelength). Images were spatially cropped to include only the relevant region of the brain. Four animals were unable to produce a suitable response within three sessions and were excluded.

### Noise and Disturbance Quantification

Information about the electrical noise was obtained by imaging a stationary object, eliminating the impact of biological disturbances and artefact components. A piece of fabric was placed in focus under the camera setup and imaging was carried out as though there was an animal present. The relative contribution of biological disturbances was determined by positioning a mouse under the camera setup and imaging the cortex without the presence of any external stimuli. Lastly, to understand the relative magnitude of a response compared to the unwanted biological disturbances, another imaging experiment was carried out with the presence of stimuli. A 10-second window of each imaging session was used to visualise the electrical, biological and response contributions. To reinforce the relative contribution of the noise and periodic artefact sources on the IOS data as well as their frequencies, Fourier analysis was carried out on this data to produce three frequency domain plots.

### Post-Processing Method

The post signal processing method consisted of two parts: the signal processing technique used on the raw data to improve the signal-to-noise ratio and the IOS analysis used to convert the raw data into the response changes in oxygenated and deoxygenated haemoglobin. All post-processing was done using MATLAB.

**Figure 1:**
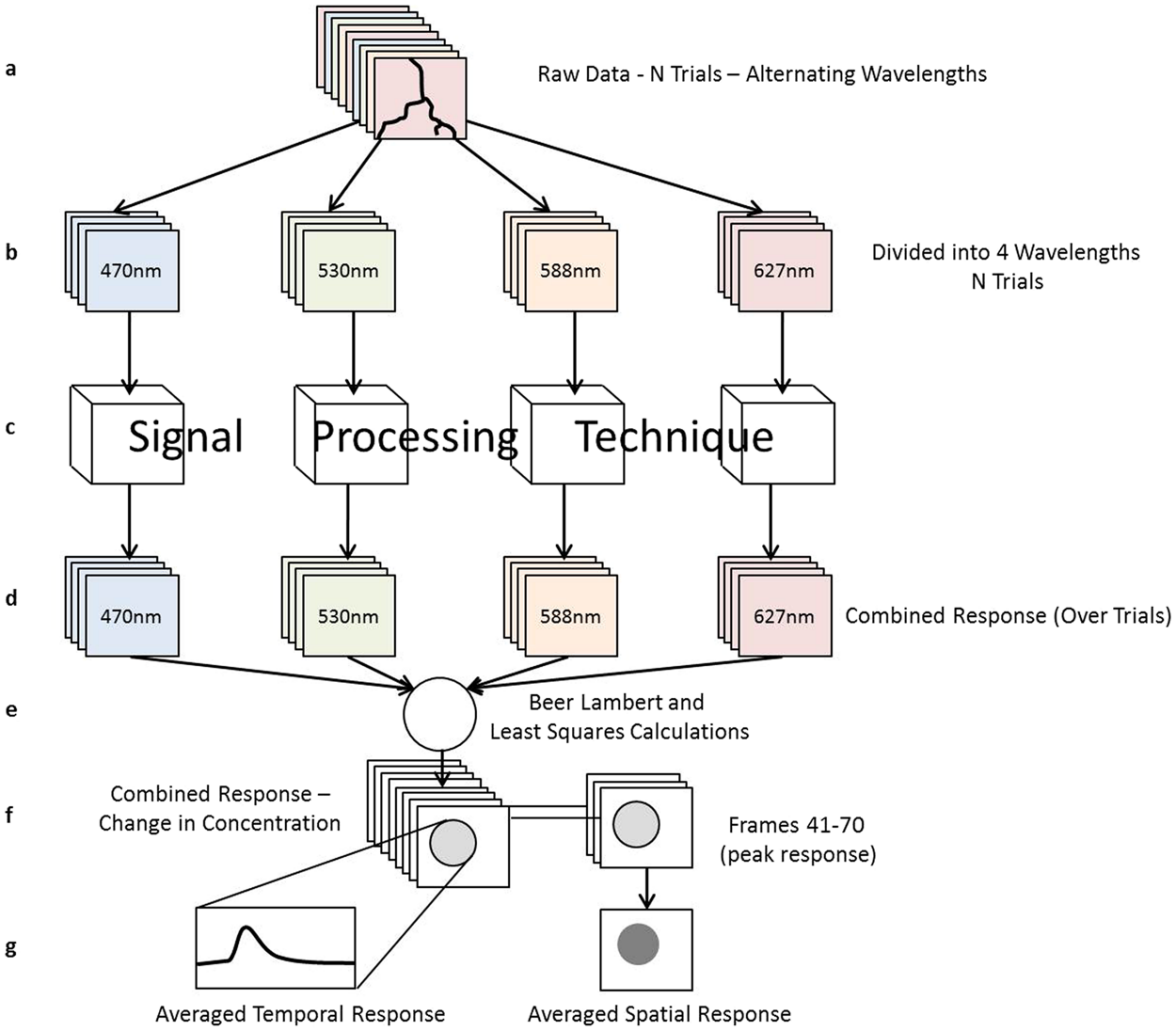
Flow chart explaining the process involved to carry out the IOS post-processing. The steps are labelled (**a**) through to (**f**).

Figure 1 displays the steps taken during the post-processing procedure. The raw data, shown in (a), was obtained from the camera in the form of x-by-y-by-t matrices, where x and y were spatial dimensions, or frames containing 260 by 280 pixels, and t was the time dimension with a resolution of 60 frames per second. For *N* trials, with 10 seconds each trial, the total matrix had a size of 260-by-280-by-600*N*. Each frame of this data contained brain images illuminated with one of the 4 wavelengths. This matrix was then divided into four new matrices, each containing data from a single illumination wavelength (b). Each resulting raw data matrix had a size of 260-by-280-by-150*N*, containing all trials. The signal processing techniques, whose suitability for IOS imaging are being compared in this paper, were performed on each of these raw data matrices separately as shown in (c). They reduced the size of the data from all *N* trials into a single trial. The resulting output was four 260 × 280 × 150 matrices (d). The post-processing techniques were performed on subsets of the raw data, containing 1 trial, 10 trials, 25 trials as well as the full 50 trials to evaluate the efficiency of each technique for varying data sizes (not shown in Fig. 1).

The next IOS analysis step converted this raw data into changes in oxygenated and deoxygenated haemoglobin, shown in (e). This process was carried out by solving the over determined system using the modified Beer-Lambert law^14^ and least squares. The equation, which relates changes in absorbance to changes in concentration, was applied to each time point of the IOS imaging data for each wavelength and is shown below,

1 $${\rm{\Delta }}A=\varepsilon l{\rm{\Delta }}C={\mathrm{log}}_{10}\,(\frac{{I}_{a}}{{I}_{0}})$$

where *ε* is the molar absorptivity, *l* is the Path length^14, 37^, Δ*C* is the change in molar concentration compared to baseline measurement, *I*_*a*_ is the post stimuli light intensity, and *I*_0_ is the baseline light intensity. Molar absorptivity values were obtained from ref. 38.

The over-determined system, created by imaging the brain under four wavelengths of light, allowed the concentrations of both HbO_2_ and HbR to be distinguished by applying least squares to the following relationship:

2 $$[\begin{array}{c}{\rm{\Delta }}{A}_{1}\\ {\rm{\Delta }}{A}_{2}\\ {\rm{\Delta }}{A}_{3}\\ {\rm{\Delta }}{A}_{4}\end{array}]=[\begin{array}{cc}{\varepsilon }_{O1}{l}_{O1} & {\varepsilon }_{R1}{l}_{R1}\\ {\varepsilon }_{O2}{l}_{O2} & {\varepsilon }_{R2}{l}_{R2}\\ {\varepsilon }_{O3}{l}_{O3} & {\varepsilon }_{R3}{l}_{R3}\\ {\varepsilon }_{O4}{l}_{O4} & {\varepsilon }_{R4}{l}_{R4}\end{array}]\times [\begin{array}{c}{\rm{\Delta }}{C}_{O}\\ {\rm{\Delta }}{C}_{R}\end{array}]$$

where the subscripts 1, 2, 3, 4 corresponded to each wavelength, and the subscripts O and R represented HbO and HbR respectively. The output of these calculations was two 260 × 260 × 150 matrices, containing the space-time response for both oxygenated (Δ*C*_*O*_) and deoxygenated haemoglobin (Δ*C*_*R*_) changes. These were summed together to produce the change in total haemoglobin (HbT) space-time response.

This space-time response was visualised as either a temporal response, displaying how the region responds through time, or a spatial response, displaying the shape and size of the response region. To produce a spatial map of the response, 30 frames, centred on the peak of the time response (frames 41–70), were averaged together, called temporal averaging, into a single 260 × 260 image. The temporal response was created by averaging a 50 × 50 pixel area, called spatial averaging, centered on the peak response area, through time into a single 150 time-point temporal signal. These two steps are shown in Fig. 1(f).

### Signal Processing Techniques

There were a variety of signal processing techniques that could be used on the raw colour data to improve the signal-to-noise ratio of the results. The following techniques were performed for each mouse that was imaged. Signal averaging over trials, temporal low pass filtering, temporal band pass filtering, principal component analysis, truncated difference and global signal regression all aimed to produce quality temporal responses as well as spatial responses from the raw mouse imaging data. Spatial Gaussian filtering was focused on producing spatial maps of the response region.

#### Signal Averaging Over Trials

The repeated stimulus trials were averaged together to produce a space-time response. Specifically, for the input signal matrix *C*_*n*_(*t*) with *n* being the trial number and *t* the time point within that trial, the average response, *R*(*t*) after *N* trials, was given by

3 $$R(t)=\frac{1}{N}\sum _{n=1}^{N}\,{C}_{n}(t)$$

#### Temporal Low Pass Filtering and Averaging Over Trials

We implemented a temporal low pass filter which attenuates signal components with higher frequencies and allows signal components with lower frequencies to pass. There were significant periodic sources of interference in the 1.5–6 Hz range of the temporal IOS imaging signal due to the biological heartbeat and breathing, whose magnitudes were similar or often greater than the signal. These periodic interferers were visualised using the noise analysis described previously. Temporal low pass filtering offered a possible way of removing this periodic interference. Each pixel of the *N* continuous trials was filtered through time using a Butterworth filter with an order of 10 and cut-off frequency of 0.5 Hz, just below the bulk of the periodic interference. Each trial in the data was then averaged together to produce a space-time response, as described previously.

#### Temporal Band Pass Filtering and Averaging Over Trials

Temporal band pass filtering was performed to remove signals with higher frequencies as well as signals of a very low frequency, including any offset component. This allowed the removal of periodic interference as well as any slow drift artefacts within the IOS data. Each pixel of the *N* continuous trials was filtered through time using a Butterworth filter with an order of 20 and high cut-off frequency of 0.5 Hz, just below the bulk of the periodic interference, and a low cut-off of 0.09 Hz, just below the stimulus frequency of 0.1 Hz. Each trial in the data was then averaged together to produce a space-time response, as in described previously.

#### Principal Component Analysis and Averaging Over Trials

We carried out Principal Component Analysis (PCA) and reconstructed the data using only the correlations with high singular values. The data was decomposed into orthogonal components through time, and ranked the based on how much they contributed to the variance in the data^39^. To perform PCA in an efficient way, we subtracted the mean from each pixel, and then perform singular value decomposition. This decomposition was given by

4 $$C=USV^{\prime} $$

Here *C* was an *L*-by-*T* space-time matrix, where *L* was the total number of pixels *p* in a frame and *T* was the total number of frames in all trials. *S* is a diagonal matrix with the diagonal elements as the singular values in decreasing order and *U* and *V* are unitary matrices. The input matrix *C* was used as shown below

5 $$C=[\begin{array}{ccc}{p}_{1,1} & \ldots  & {p}_{1,T}\\ \vdots  & \ddots  & \vdots \\ {p}_{L,1} & \ldots  & {p}_{L,T}\end{array}]$$

The desired temporal signal was extracted by reconstructing *R* = *USV*′, using only the correlations with high singular values. The assumption was that lower ranked values are composed primarily of uncorrelated disturbances.

The cut-off for the component selection was decided by visually inspecting a plot of the eigenvalues of the components plotted against their corresponding component number (scree plot), choosing a cut-off just past the elbow of the scree plot. Typically, keeping the first 25% of components was suitable. The reconstructed data was be the same length as the un-processed data. Averaging over trials was therefore needed to be carried out to produce the space-time response.

#### Truncated Difference and Averaging Over Trials

A variation of PCA was performed, called truncated difference, which uses a different method for selecting which components to keep when reconstructing the signal. Each principal component was compared to a stimulus presentation sequence and a correlation value between the two was calculated. Components were then selected based on this value, with higher valued components being more likely to contribute to the stimulus response. A more detailed explanation of this technique can be found in ref. 30. In our implementation, we used a stimulus presentation sequence defined as

6 $$w(t)=\{\begin{array}{cc}-1 & {\rm{f}}{\rm{o}}{\rm{r}}\,1\le t\le 22\\ 0 & {\rm{f}}{\rm{o}}{\rm{r}}\,23\le t\le 40\\ 1 & {\rm{f}}{\rm{o}}{\rm{r}}\,41\le t\le 70\\ 0 & {\rm{f}}{\rm{o}}{\rm{r}}\,71\le t\le 150\end{array}$$

repeated temporally for each stimuli trial, where *t* is the frame location within that trial. That is, we selected components which most closely matched a step change in response (when compared to baseline, *t* = 1 to 22), starting at *t* = 41 and ending at *t* = 70, spanning the peak of the response. Signal values outside these intervals were not considered. We implemented a selection criteria of components whose correlation values were above 3 times the mean correlation value of all components. This removed components that are uncorrelated, and therefore assumed to be unrelated to the signal, whilst keeping those that are considered a response. The selected components were then used to reconstruct the desired signal. Each trial in the data was then averaged together to produce a space-time response, as in described previously.

#### Global Signal Regression and Averaging Over Trials

Global signal regression (GSR) was used to remove global patterns, presumed to be unimportant, from the response signals^40^. This is potentially useful for IOS imaging as the response signal was unique to an area, where as biological disturbances such as breathing and movement were most likely be global. The global signal *g*(*t*) was initially calculated by averaging the time signal of every pixel *i* of the imaging data. Regression of this signal was achieved by solving

7 $${R}_{i}(t)={C}_{i}(t)-g(t){\beta }_{i}$$

for each pixel *i*, where *R*_*i*_(*t*) was the response signal after regression, *C*_*i*_(*t*) was the measured concentration data as a column vector and *β*_*i*_ was the regression coefficient estimated by

8 $${\beta }_{i}={(g{(t)}^{T}g(t))}^{-1}g{(t)}^{T}{C}_{i}(t)$$

that is, the degree to which the global signal was removed from each pixel depends on the correlation of that global signal to that pixels time signal.

Global signal regression was performed on the whole set of imaging data, using the total average of all pixels’ time series as the global regressor g(t). The resulting matrix was averaged over trials as described previously to produce a space-time response.

#### Spatial Gaussian Filtering and Averaging Over Trials

A Spatial Gaussian filter was used to improve the spatial response maps of the data by smoothing pixels based on their surrounding pixels. Two dimensional Gaussian filtering was performed on the spatial data by convolving it with the Gaussian kernel defined by

9 $$F(x,y)=\frac{1}{2\pi {\sigma }^{2}}{e}^{-\frac{{x}^{2}+{y}^{2}}{2{\sigma }^{2}}}$$

where *x* and *y* were the pixel locations in the horizontal and vertical axis respectively, and *σ* was the standard deviation of the Gaussian distribution. A Gaussian filter (*σ* = 200 *um*) was performed on each individual frame of the raw imaging data. We found that a wide range of standard deviations (*σ*) of the filter had minimal impact on the results. The resulting output was averaged through trials to produce a space-time response.

### Estimating the Signal-to-Noise Ratio

To compare the performance of each signal processing technique, it was beneficial to quantify the quality of the results. A simple yet effective way to do this was to calculate the signal-to-noise ratio (SNR) of the resulting output of each signal processing technique. The SNR quantified the amount of useful information in a signal relative to that of unwanted disturbances and was given by

10 $${\rm{SNR}}=\frac{{P}_{{\rm{Signal}}}}{{P}_{{\rm{Noise}}}}$$

where *P*_Signal_ was the power of the signal component and *P*_Noise_ was the power of the noise component. The power of a component is given by

11 $${\rm{Power}}=\sum _{z=0}^{Z}{|R(z)|}^{2}$$

where *R*(*z*) was the response data matrix with *Z* elements.

The issue with the IOS data however is that the true signal is not known, but rather the resulting output is a sum of the signal and the noise, whose power is given by *P*_Total_. To deal with this situation, we took two equal size sets of imaging data from each mouse, labeled data1 and data2, and used these to estimate the values for signal and noise power. The reason behind using these two data sets was that the response signal should occur in both halves of the data. Any random noise would be different in each set, thus any component of the response which was the same in each half would be taken as signal. The noise power was calculated by

12 $${P}_{{\rm{Noise}}}={\rm{power}}({\rm{data1}}-{\rm{data2}}\mathrm{)/2}$$

This calculation made the assumption of a zero mean additive noise present in the signal, thus the subtraction removed common signal and doubled the noise power. The power of the total signal could be taken as the power of data1 or data2, or their average. We opted to use the average:

13 $${P}_{{\rm{Total}}}=({\rm{power}}({\rm{data1}})+{\rm{power}}({\rm{data2}}\mathrm{))/2}$$

Finally, the signal-to-noise ratio was estimated as

14 $${\rm{SNR}}=\frac{{P}_{{\rm{Signal}}}}{{P}_{{\rm{Noise}}}}=\frac{{P}_{{\rm{Total}}}-{P}_{{\rm{Noise}}}}{{P}_{{\rm{Noise}}}}$$

These calculations were performed on both the spatial and temporal output response for each animal for each signal processing technique.

## Results

### Noise and Disturbance Analysis

**Figure 2:**
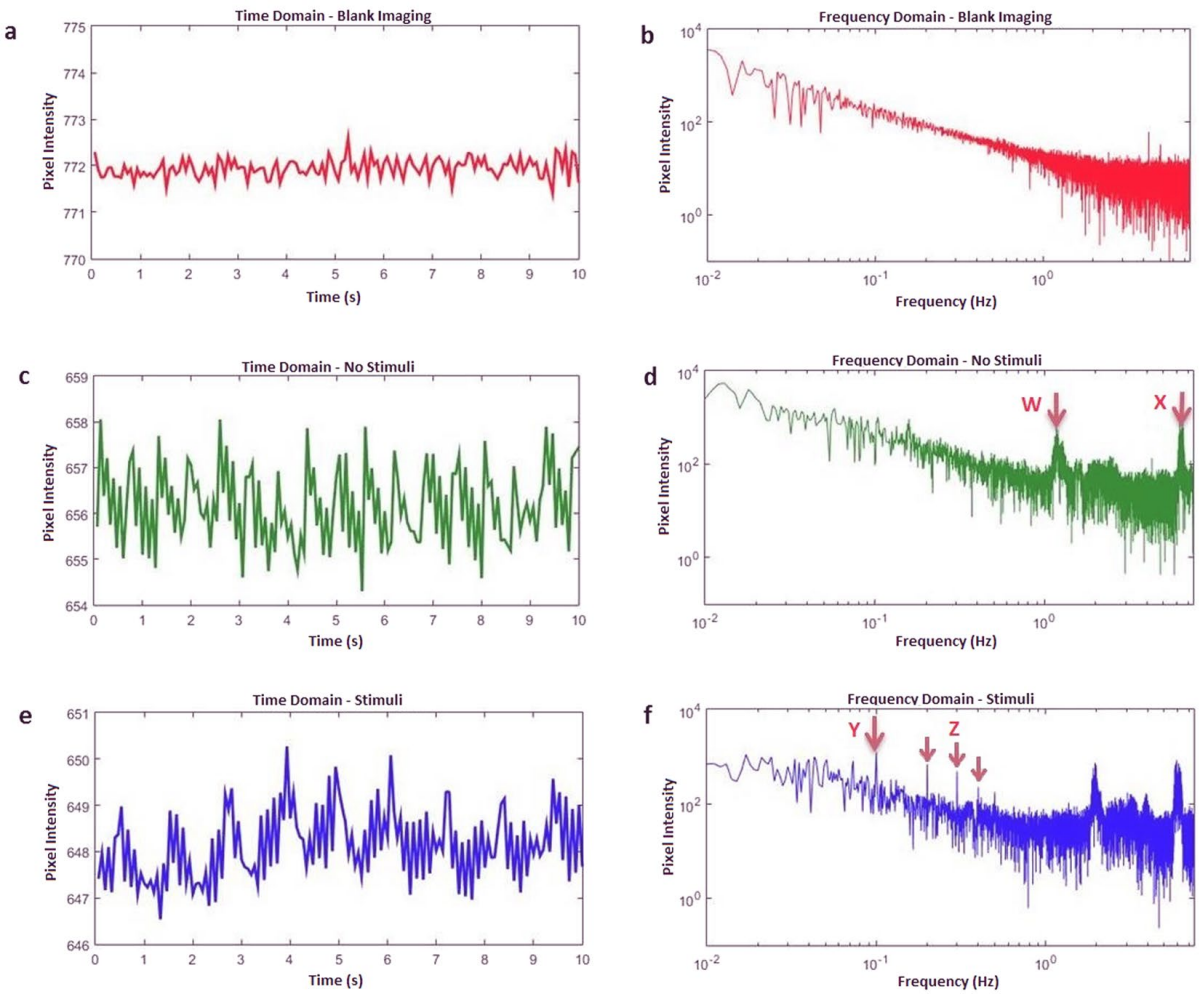
The relative impacts of biological and electrical disturbances. (**a**) Blank imaging data representing the electrical disturbances of the system, obtained by imaging a non-biological surface for a single trial. All time signals have been plotted with the same axis range for ease of comparison. (**b**) Frequency response of the electrical noise. (**c**) Biological disturbances obtained from imaging the brain over a single trial without stimuli. (**d**) Frequency response without stimuli. The peaks at (W) and (X) are the result of breathing and heartbeat respectively. (**e**) Single trial response obtained by imaging the brain with stimuli. (**f**) Frequency response with stimuli. The peak at (Y) is the result of the response due to the stimuli, which has a 10-second period. Several harmonic components (Z) of this signal are also present.

The relative impact of biological and electrical disturbances can be seen in Fig. 2. For our setup, biological disturbances have a far larger contribution to the signal than electrical disturbances. This is highlighted in Fig. 2(a,c), by comparing the relatively larger magnitude of the no stimuli imaging (representing biological artefacts) to that of blank imaging (representing electrical noise). It is also evident that there is a periodic component present in the biological disturbances. A greater understanding of the frequency components of this periodic interference can be obtained by focusing on Fig. 2(d). The peaks at 1.2 Hz (labeled W) and 6 Hz (labeled X) are the result of breathing and heartbeat respectively, with their harmonic components also present, although less visible in this example. Figure 2(e) shows a single trial of the mouse brain with stimuli. The signal primarily consists of disturbances and the presence of a response is barely, if at all, apparent. The frequency domain of this signal, Fig. 2(f), shows a peak at 0.1 Hz (labeled Y). This is the result of the response due to the 10-second stimulus period. This 0.1 Hz peak is not present in areas of the brain not affected by stimuli, or in the no stimuli case presented. Although the magnitude of the 0.1 Hz response is obviously larger than its immediately surrounding frequencies, it is still comparable in magnitude to that of the biological disturbances. This highlights why without proper signal processing, the IOS response will not be visible. It should be noted that some of these features will be unique to our imaging setup. Different cameras, illumination setups and animal species, for example, will result in different values for this disturbance quantification. Although we predict the relative contributions will remain the roughly the same, laboratories are encouraged to perform their own quantification to aid in their post processing analysis understanding.

### Signal Processing - Temporal Response

**Figure 3:**
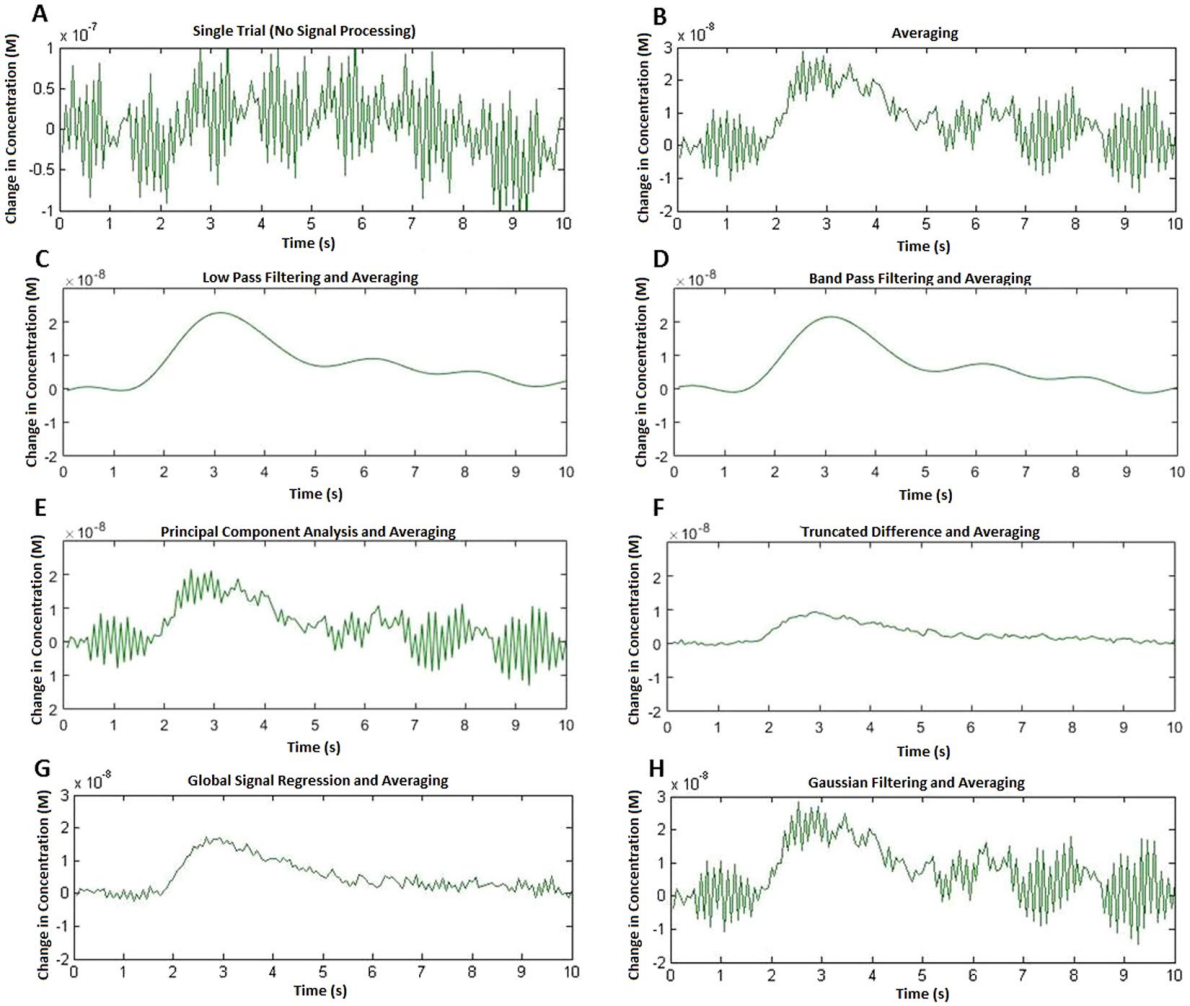
Temporal responses obtained from IOS imaging of the mouse brain in response to stimuli. Stimuli occur at 1.5 s. A 50 × 50 pixel area was selected, centered within the response region and spatially averaged together to produce the time domain signals. The responses of a single mouse, imaged for 50 trails, are shown. Note that this single example is not indicative of all group members as, is well known^35^, there is considerable within group variability for many techniques. (**A**) Shows a single trial however it should be noted that its Y-axis is different to the other plots, allowing the larger magnitude of disturbances to be seen in its entirety. (**C**–**H**) Show the effect of each signal processing technique for all 50 trials.

To understand the effects of each technique on the resulting temporal output, the responses of a single mouse are shown in Fig. 3. The first plot (Fig. 3(A)) emphasizes the relative disturbances and signal of a single trial. The following time domain plots highlight the temporal effects of each signal processing technique. Although the results vary between animals, the relative outcomes of the technique remain relatively consistent.

**Figure 4:**
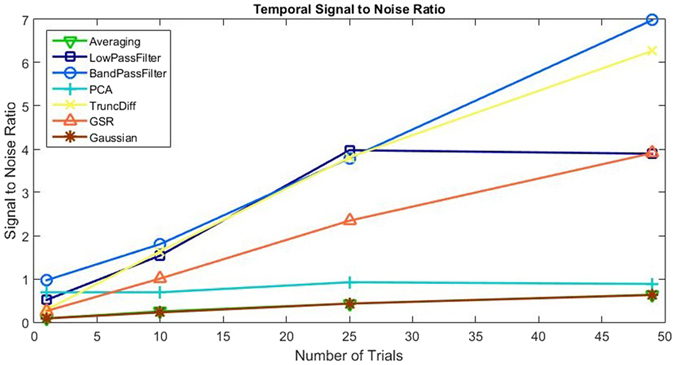
The temporal SNR of each technique is plotted against the number of trials used for calculations. SNR values were calculated as described in previously. Plotted values are taken as the average over 19 animals.

To quantify the performance of each technique at improving the IOS data, SNR values were calculated as described previously. Figure 4 shows how the SNR changes with the number of trials for each signal processing technique. The values shown are the average of all 19 animals’ SNR results.

**Figure 5:**
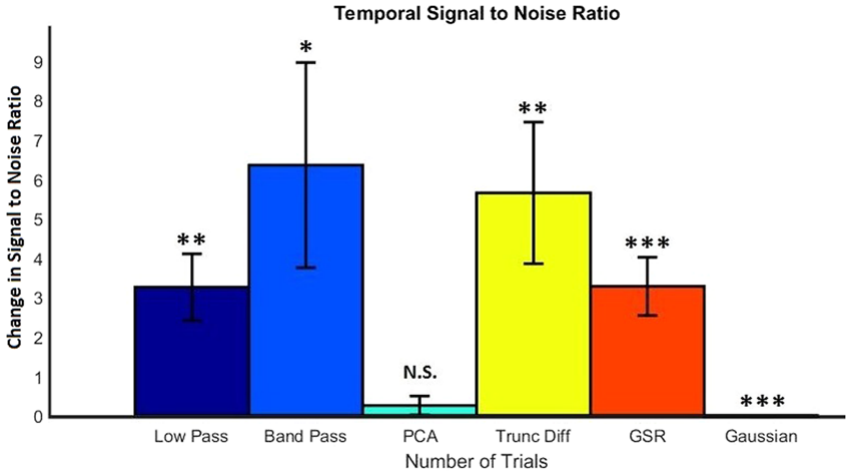
Temporal SNR - Change in SNR values compared to averaging over trials for each technique when 50 trials are used for each signal. SNR values were calculated as described previously. Values are taken as the average of 19 animals and standard error bars are shown. Note that the error bars reflect the large within group variability for many of the techniques^35^. Gaussian filtering produced a significant but extremely small decrease in SNR compared to averaging. Paired T-Test was performed on the SNR values compared to averaging over trials, *p < 0.05, **p < 0.01, ***p < 0.001, N.S. - not significant.

As a larger number of trials are used, the differences between the signal processing techniques become more apparent. Figure 5 presents the change in temporal SNR when compared with averaging over trials for all techniques at 50 trials. Standard error is shown for each technique. Low pass filtering, band pass filtering, truncated difference and GSR significantly outperformed that of averaging, while PCA did not significantly improve on averaging over trials. Gaussian filtering actually resulted in a decrease in SNR, however the change was very small.

Comparing the component selection for PCA to truncated difference, the elbow of the scree plot corresponded to the first 20% of the components and explained around 25% of the variance whereas truncated differences found many fewer components to be correlated with the stimuli, selecting around 1.5% of the components corresponding to around 1.5% of the variance.

### Signal Processing - Spatial Response

**Figure 6:**
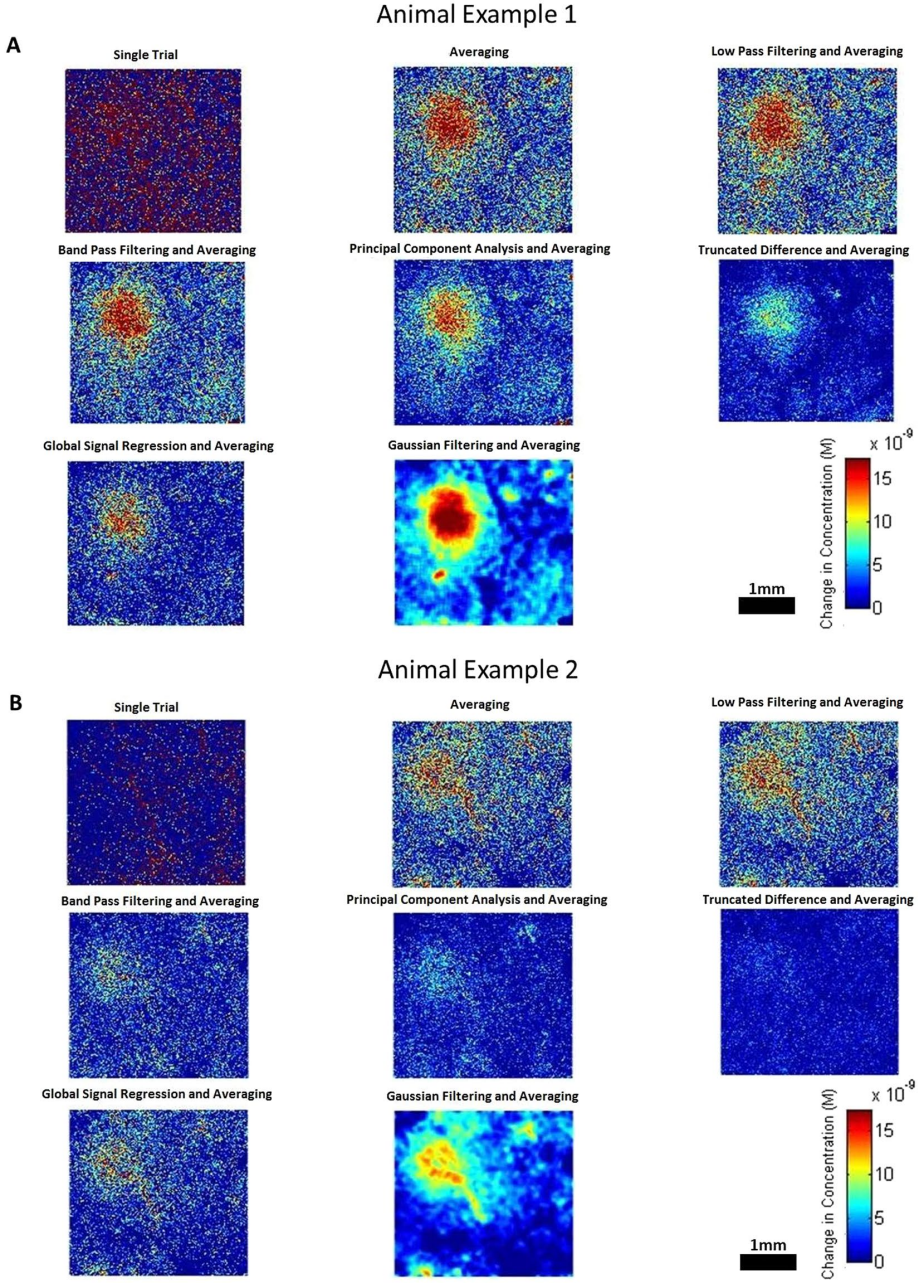
Spatial responses obtained from IOS imaging of the mouse brain in response to stimuli. Response examples are shown from two different animals, labeled (**A**,**B**). Each image shows the performance of each technique on producing a spatial response for 50 trails. Note that two examples are shown to highlight the considerable within group variability for many techniques and serve as only an example to the performance of each technique across all animals. Frames were temporally averaged between the 2 s and 3.5 s time points to produce a single response map.

To understand the effects of each signal processing technique on the resulting spatial output, the responses of two mice have been shown in Fig. 6. The spatial maps highlight the performance of each technique on producing a clear spatial response. These responses serve only as an example as to the effects of each processing technique as well as how they may vary between animals.

**Figure 7:**
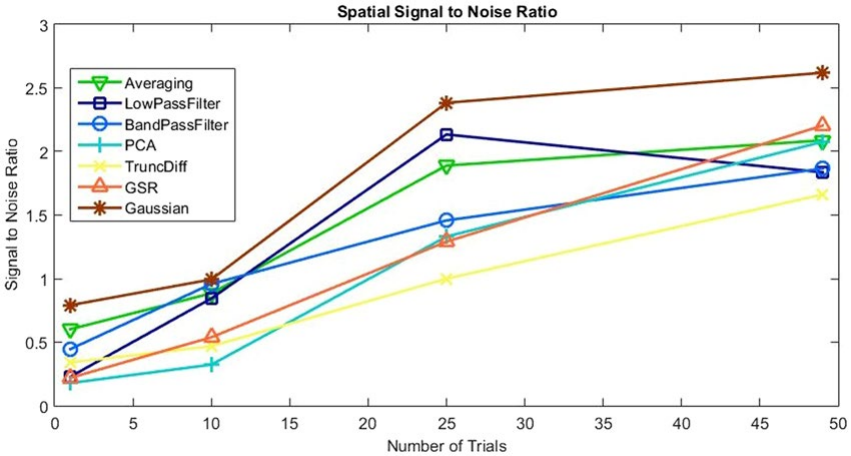
The spatial SNR of each technique is plotted against the number of trials used for calculations. SNR values were calculated as described previously. Values plotted are the average of 19 animals.

To quantify the performance of each technique at improving the IOS data, SNR values were calculated as described previously. Figure 7 shows how the SNR changes with the number of trials for each signal processing technique. The values shown are the average of all 19 animals’ SNR results.

**Figure 8:**
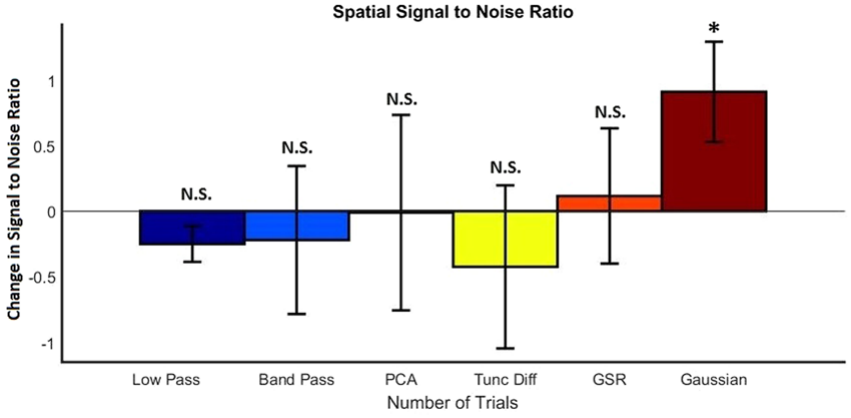
Spatial SNR - Change in SNR values for each technique compared to averaging over trials when 50 trials are used for each signal. SNR values were calculated as described previously. Values are the average of 19 animals and standard error bars are shown. Paired T-Test was performed on the SNR values compared to Averaging over trials, *p < 0.05, **p < 0.01, ***p < 0.001, N.S. - not significant.

Figure 8 presents the change in spatial SNR compared to averaging over trials for all techniques at 50 trials. Standard error is shown for each technique. Only Gaussian filtering performed significantly above averaging. There was significant variability in the performance of the other techniques, as highlighted by the large amount of error.

## Discussion

Of the many IOS post-processing options available, averaging over trials is the most computationally simple technique to implement. Despite the widespread use of averaging, a variety of other techniques are available that may be capable of delivering better results than those provided by averaging alone. However a direct head to head comparison of techniques is not available. As such, we were motivated to directly examine a set of well understood signal processing algorithms in the current paper. The primary metric we used for comparison was the signal to noise ratio. The SNR, which compares the power of the signal to the power of the noise, provides a simple method to compare the accuracy and efficiency of signal processing approaches.

In applying the SNR comparative approach we recognised that there are two quite distinct endpoints for IOS imaging processing; the first being a temporal response and the second spatial response. The SNR metric was useful for evaluating the comparative performance of both of these. Our results indicated that there are considerable differences between the SNR performances of each of the signal processing analyses assessed. We found, as expected, that averaging over trials was, in a number of instances, not the best performing technique. Indeed, for temporal performance, low pass filtering, band pass filtering, truncated difference and GSR provided an improved SNR in our context, whilst Gaussian filtering outperformed averaging alone in the spatial context. The following paragraphs will provide a detailed overview of the performance of each individual technique and then conclude with a set of recommendations that could be used by those undertaking IOS imaging in the future to better help decide which signal processing technique evaluated in this study would best suit their needs.

As stated previously, averaging over trials is the simplest and most commonly used technique for IOS imaging. It is therefore useful to consider averaging as the ‘baseline’ or benchmark technique. Averaging’s temporal performance, shown by our SNR calculations in Fig. 4, indicates a steady increase in SNR as number of trials increases, however the example shown in Fig. 3 shows that the contributions of the disturbances is still clearly visible. Averaging over trials relies on disturbances being random with zero mean and therefore unrelated to the response signal. As established previously however, the most significant source of disturbances is composed of periodic biological interference. Periodic disturbances can have a detrimental effect on the averaging over trials result, depending on its timing relative to the stimuli. For the example chosen, this was not a significant issue, however if the periodic interferers’ frequencies had been a multiple of the stimulus frequency, averaging over trials would amplify the interference as well as the signal. If this occurs, one option is to repeat the imaging with a different stimuli period. Another, which we consider here, is to improve the signal processing technique.

Both forms of temporal filtering investigated, low pass and band pass, significantly improved the temporal IOS signal. As expected, the SNR improvement steadily increases as the number of trials increased. They also produced the smoothest response for the example signal, as shown in Fig. 3.

Low pass and band pass filtering have several limitations in their application. Firstly, they require periodic stimuli to be performed, which could be an issue if the IOS setup and form of stimuli does not easily allow for this, for example, stimuli timed to an external or uncontrollable event. Low pass filtering removes high frequency components from the signal, such as breathing and heartbeat, which produces the smoothest of the signal processing responses, but also removes any steep or rapid changes that may actually be a part of the desired response. For the typical IOS response this will not be the case, however depending on the application, this should be considered if sharp changes in the temporal response are the focus of the experiment. Band pass filtering removes low frequency components as well as those of high frequency. This is useful for removing any slow drift artefacts that may be present in the signal, for example, from changes in anesthesia depth to unstable electrical components. As with low pass, this also has the potential to remove signal that may be valuable. Care should be taken when selecting an appropriate filter, ensuring cut-off values successfully remove the periodic interferes without disrupting the required IOS response. Figure 4 shows that lowpass filtering has a drop in performance between 25 and 50 trials, whilst bandpass continues to perform well. This is due to an increasing global drift in the data over the last few trials, which low pass filtering is unable to remove. Other techniques, such as band pass filtering, GSR and truncated difference are capable of dealing with this drift and therefore their SNR values also improved.

GSR produces SNR values similar to that of temporal low pass filtering if a large number of trials are used. However, it does not require educated decisions to be made during implementation. GSR excels at displaying changes that are unique to the response region and eliminating any significant global changes that are an issue, such as heartbeat and breathing. However, GSR produces a dampening effect on the magnitude of the signal, reducing the SNR^40^. For this reason, GSR has caused significant controversy for functional connectivity analysis, although it is recognised to be a useful method to remove correlations in stimuli-response measurements such as ours performed here^41^. GSR should be considered for improving the temporal response signal if performing periodic stimulus is not possible or if there are global disturbances at frequencies very close to the stimuli frequency.

Spatial Gaussian filtering did not produce any significant improvement to the resulting temporal output when compared with averaging over trials. The statistically significant, yet marginal, decrease in SNR due to Gaussian filtering is due to the fact the signal gets spread out to surrounding pixels, reducing its magnitude within the region of interest. This reduces the utility of any additional spatial filtering, such as Gaussian filtering, for temporal data. Gaussian filtering does however have a significant impact on the spatial SNR, which will be discussed later.

Principal component analysis did not improve the temporal response when compared to averaging, primarily due to two main issues. Firstly, choosing a cut-off point slightly higher than the elbow of the scree plot enables the bulk of the signal components to be kept and removes many noise components, however, this selection will still contain a variety of interference components. Components with high variance will not be removed, in fact the components with the largest variance are often global heartbeat and breathing interferes. Secondly, PCA excels when there is a large difference between the variance of the disturbance and signal components, unfortunately this is not the case for our data and the response signal can be found in components well past the elbow of the scree plot. These response signals will therefore be removed, reducing signal strength as well as the SNR. Correct component selection is therefore vitally important for PCA based analysis.

Truncated difference attempts to resolves the component selection issues encountered with PCA. The technique will prioritise components which have a change from baseline directly following stimuli and discarding those components (due to interferers) which may have a large variance but do not correlate with the stimuli. The choice of which components have a large enough correlation score is not always straightforward, but for our data, selecting components with at least 3 times the mean correlation score worked well, performing comparably to temporal bandpass filtering, to produce a large increase in SNR compared to averaging. The improvement of truncated difference over PCA is due to the exclusion of high variance components unrelated to the stimuli.

When there is a particular interest in the spatial composition of the response, it is necessary to reconsider the effects of each technique in terms of spatial SNR. As stated earlier, averaging over trials relies on the noise of the data being random with a zero mean and unrelated to the response signal. For the spatial noise in our experiment, this is typically true, and averaging over trials greatly increases the SNR as the number of trials increase. Spatial data can contain significant artefacts resulting from changes in vascular architecture or movement and averaging over trials is usually successful at dampening these artefacts, although they will not be entirely removed.

By observing the example responses in Fig. 6, it can be seen that there is still a large amount of pixel noise present in the images. This suggests that a spatial filter, such as Gaussian filtering, would be useful. A Gaussian filter, which smooths Gaussian noise within pixels, significantly increases the SNR of the spatial data. This image also visually looks much more appealing, which is beneficial for more easily visualising results. Gaussian filtering does potentially remove sharp spatial changes which could produce a loss of detail in the resulting data which should be considered.

The remaining techniques, low pass filtering, band pass filtering, GSR, PCA and Truncated Difference, did not show any significant improvement over averaging through trials alone for the spatial response data. The relatively large error bars shown in Fig. 8 suggests the performance of these techniques vary considerably. Figure 6 highlights this point, showing two examples, one where the techniques perform well (A) and another where they perform considerably worse (B). Gaussian filtering however is a consistent improvement over averaging. While it is not recommended that Gaussian filtering be performed for improving the SNR of temporal IOS data it should be noted that since the calculations are typically performed post hoc, there is no reason one couldn’t carry out one technique for temporal results, and another for spatial, thus the spatial benefits of Gaussian filtering, for example, can be obtained without its corresponding negative temporal impacts.

Despite our results suggesting that PCA is not useful for spatial data, many groups who implement IOS imaging have shown apparent success with different variations of the technique. They often do not use PCA to remove noise, but rather to separate multiple components, as is usually the case with visual stimuli orientation mapping, or to remove any specific unwanted disturbances. We have not assessed the techniques’ performance for these cases in this paper. Regardless of the purpose of performing the PCA based technique, component selection is extremely important, as highlighted in the difference in results between PCA and truncated difference for temporal data. Whilst PCA selects the first set number of components and truncated difference chooses components based on correlation to the stimuli presentation, several other variations exist for component selection. Components can be manually selected, based on visual inspection, which could be time consuming or produce significant user bias. The indicator function method includes the same components as PCA but scales each components based on its correlation with the stimuli^30, 33, 34^. For our stimuli, the end result of applying the indicator function method is that each PCA component is scaled by a factor inversely proportional to the variance of that component. This would improve on traditional PCA if the signal components had lower variance than the interference components, however this is not necessarily the case and this scaling can have the effect of scaling up some low variance interference components producing a poor result. Blind source separation also uses PCA, however the components are assumed to vary smoothly across space and different components are assumed to be spatially uncorrelated^31^. The components are recombined into separate spatially uncorrelated source components. This can work well if the both the signal and the interferers are spatially uncorrelated, such as in visual stimuli orientation maps, however this is not true for our single physical stimuli case.

Kalatsky and Stryker have proposed an alternative technique for producing the spatial response of the visual cortex with periodic stimuli^28^. By stimulating with a specific stimulus frequency, the response approximates the form of a sine wave of that frequency. The frequency response of each location in the brain shows that pixels within the response region will have a large peak at the stimulus frequency, whereas areas outside the region will not have this peak. Each pixel is then assigned a value equal to its stimulus frequency response value to create the spatial response map. This has potential to produce improved spatial response, however for our stimulus modality and frequency; the stimuli does not closely resemble that of a sine wave, making this technique not feasible.

Imaging setups can vary widely between research groups so it will be beneficial to comment on what effect these differences might have on our results and the choice of post-processing method. Some variables of the setup are easy to predict. For example: A different camera setup will impact on the results if electrical noise is increased. Other animal species will have different heart rate and breathing rates, which will have an impact on the choice of filtering parameters. Clearer or more opaque imaging windows to the brain may increase or decrease the camera’s detected signal strength but we expect should have an equal effect on the signal processing methods. Imaging setups that investigate other stimulus modalities may produce a response with a different shape or magnitude, or perhaps multiple response regions, as with visual orientation maps. One major difference between imaging setups is the existence of multiple wavelength imaging versus a single illumination setup. The outputs of our 4 wavelength and the alternative 1 wavelength methods are different (One measures the change in haemoglobin concentration the other is change in reflectance), meaning the comparison between the two is not straightforward. Imaging with multiple wavelengths gives more information about oxygen changes, rather than attempting to improve the SNR. While using 4 wavelengths instead of the minimum 2 can improve the SNR by using least mean squares, it is not clear that this is more beneficial to the SNR than imaging at 2 wavelengths twice as often and averaging to remove noise. Nonetheless the results of our paper should translate directly across to the single wavelength case as the relative improvement of each technique should remain similar.

Another comment that should be made is in regards to the effectiveness of SNR as a means to compare the performance of the techniques. The fact that a true IOS response is not known is a source of difficulty for studies such as this one, meaning a true calculation of SNR would not be possible, as it requires the true signal to be known. Our SNR estimation attempts to resolve this by dividing the data into two sets. The response signal should occur in both halves of the data. Any noise is random and will be different in each set, thus any component of the response which is the same in each half will be taken as signal. For ideal cases, this works well, however there are some unique cases where this may give an inaccurate estimate. For example, a large amplitude artefact that is present in both halves could be mistaken as signal. Although these issues will be present across all techniques, since they are performed on the same data, it could create bias towards some techniques over others. SNR also fails to consider other qualities of the techniques, such as separating multiple responses.

For a given technique, when large within group variability is present, it may often be possible to find a single case where it performs well even though, on average, its performance is poor. This emphasises the importance of attempts to provide quantitative rather than qualitative comparison techniques. Future investigation into IOS imaging techniques could include SNR values for comparison, and also implement other ground truths for the true signal, such as multiple region stimulation and calcium imaging.

In conclusion, Gaussian filtering is the most effective choice for improving the spatial IOS response with minimal complexity. For temporal data, low pass filtering and band pass filtering provide a significant improvement over averaging with reasonable complexity, however if periodic stimuli cannot be used, GSR or truncated difference should be considered as these techniques also significantly improve the signal and do not require a periodic stimuli. Other PCA variations can be considered but require careful consideration of the component selection technique to be used.
